# OpenMPIData: An initiative for freely accessible magnetic particle imaging data

**DOI:** 10.1016/j.dib.2019.104971

**Published:** 2019-12-11

**Authors:** Tobias Knopp, Patryk Szwargulski, Florian Griese, Matthias Gräser

**Affiliations:** a Section for Biomedical Imaging, University Medical Center Hamburg-Eppendorf, Germany; b Institute for Biomedical Imaging, Hamburg University of Technology, Germany

**Keywords:** Magnetic particle imaging, Magnetic nanoparticles, MDF, HDF5

## Abstract

Magnetic particle imaging is a tomographic imaging technique capable of measuring the local concentration of magnetic nanoparticles that can be used as tracers in biomedical applications. Since MPI is still at a very early stage of development, there are only a few MPI systems worldwide that are primarily operated by technical research groups that develop the systems themselves. It is therefore difficult for researchers without direct access to an MPI system to obtain experimental MPI data. The purpose of the OpenMPIData initiative is to make experimental MPI data freely accessible via a web platform. Measurements are performed with multiple phantoms and different image sequences from 1D to 3D. The datasets are stored in the magnetic particle image data format (MDF), an open document standard for storing MPI data. The open data is mainly intended for mathematicians and algorithm developers working on new reconstruction algorithms. Each dataset is designed to pose a specific challenge to image reconstruction. In addition to the measurement data, computer aided design (CAD) drawings of the phantoms are also provided so that the exact dimensions of the particle concentrations are known. Thus, the phantoms can be reproduced by other research groups using additive manufacturing. These reproduced phantoms can be used to compare different MPI systems.

**Table:** Specifications Table

Subject	Radiology and Imaging
Specific subject area	Magnetic Particle Imaging: A tomographic imaging technique capable of imaging magnetic nanoparticles.
Type of data	Voltage time series, CAD models
How data were acquired	The data were acquired with a preclinical MPI scanner (Bruker, Ettlingen).
Data format	Datasets are stored in the Magnetic Particle Imaging Data Format (MDF). The specification can be found at https://arxiv.org/abs/1602.06072 Particle phantom CAD data are available in various data formats (SolidWorks part file, STL file, STEP file)
Parameters for data collection	Data were measured with three different particle phantoms (shape phantom, resolution phantom, concentration phantom) and with three different imaging sequences (1D Cartesian sequence, 2D Lissajous sequence, and 3D Lissajous sequence). In addition, we measured for each imaging sequence a dedicated calibration dataset that can be used to setup the MPI system matrix.
Description of data collection	First, the phantoms were constructed using a CAD program (SolidWorks) and 3D printed with a stereolithography printer (Form2, Formlabs). Afterwards, the phantoms were impregnated (NanoSeal, JELN, Schwalmtal, Germany) to avoid liquid absorption by resin material. Then, the phantoms were filled with the fluid tracer perimag (micromod, Rostock) that contains magnetic nanoparticles generating the signal in MPI. The phantoms were mounted on a robot that positioned the phantoms at the center of the scanner bore. After performing the measurements with the system software ParaVision, the measurement data were available in a proprietary data format from the vendor Bruker. We then converted the data into the open MDF data format using the open-source software MPIFiles.jl.
Data source location	Institution: University Medical Center Hamburg-Eppendorf City: Hamburg Country: Germany Latitude and longitude (and GPS coordinates) for collected samples/data: 53.596977,9.973756 (53° 35′ 49.1172″ N 9° 58′ 25.5216″ E)
Data accessibility	Repository name: OpenMPIData.jl Direct URL to data: https://github.com/MagneticParticleImaging/OpenMPIData.jl The documentation of the data is hosted in a Github repository that can be accessed through the above link.

**Table:** 

**Value of the Data** • The open MPI datasets are the first datasets that are freely accessible to the research community. Since the number of MPI scanners installed worldwide is limited, it is not simple to obtain MPI datasets for research groups that do not develop or operate an MPI scanner but want work on new reconstruction algorithms. Datasets are often only accessible by cooperation with a research group and accepting certain research agreements. In order to compare new reconstruction approaches it is, however, essential to have reference datasets that can be used freely by any researcher worldwide. Beside the data itself, it is also important to have a detailed description how the data was measured in order to reproduce the experiments. In case of the tomographic object measurements, a geometric description of the object is essential, which allows comparing reconstructed data with a ground truth. All this is provided by the open MPI datasets, which makes them useful for various research groups. The data has already been adopted by researchers and e.g. used in Ref. [1]. • Two different kinds of groups can benefit from this data. First, mathematical/algorithmic researchers developing reconstruction algorithms that do not have access to an MPI scanner. Second, MPI researchers can benefit from the data since they form reference datasets, to which future measurements can be compared to. • The data can be used in two different ways. First, the data can be used to apply custom image reconstruction methods and comparing their results with the provided reference reconstruction. Second, other research groups can reproduce the phantoms using a 3D printer and the provided CAD files to compare their MPI scanner by performing measurements with the reproduced phantoms.

## 1 Data

**Fig. 1:**
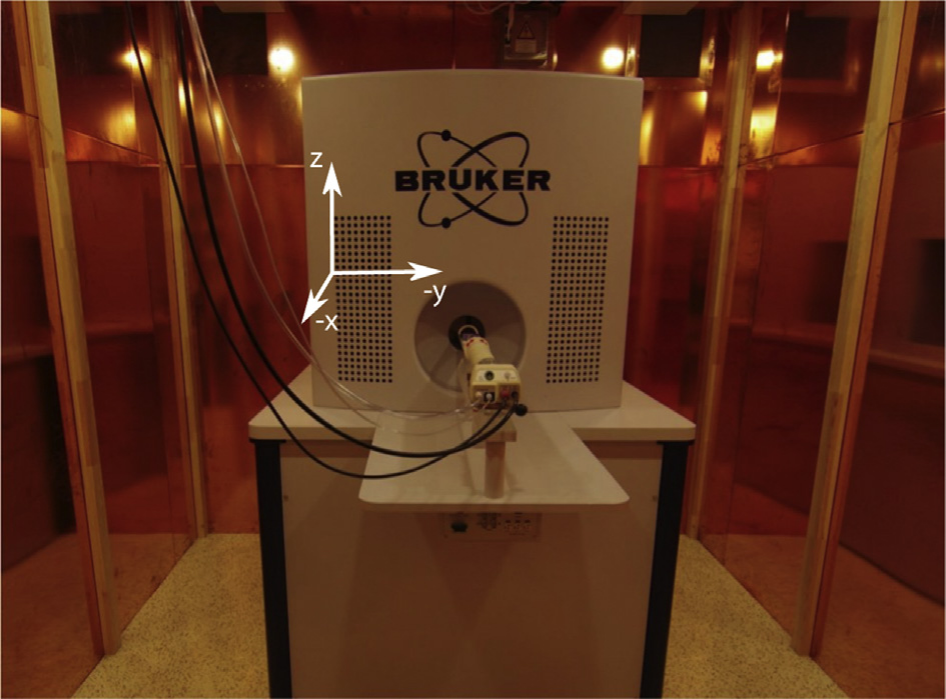
Preclinical MPI scanner used for the measurements.

In the following subsections we outline the phantoms and parameters that were used to acquire the magnetic particle imaging (MPI) [2] datasets. All measurements were performed using the preclinical MPI scanner shown in Fig. 1. In all descriptions we refer to the coordinate system shown the figure.

### 1.1 Phantoms

**Fig. 2:**
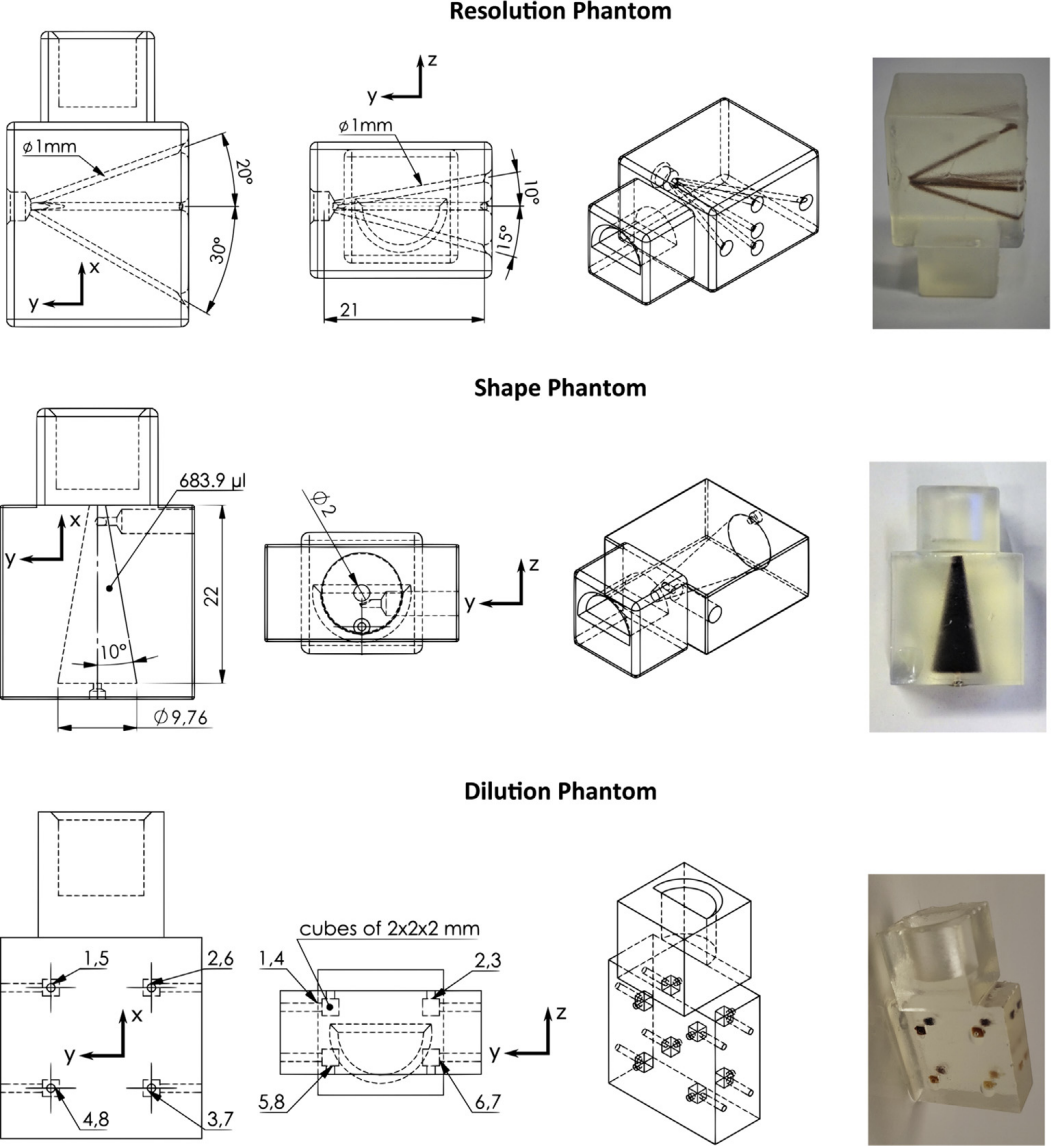
Phantoms used in the open MPI datasets.

The measurements were performed with three different object phantoms. In all cases the tracer perimag (micromod, Rostock) was used for contrast generation. Pictures of the three phantoms are shown in Fig. 2.

The first phantom is a cone having a tip radius of 1 mm, an apex angle of 10° and a height of 22 mm. The total volume of the cone is 683.9 μL. The tracer concentration used in the experiments was 50 mmol(Fe)/L. The phantom can be rendered in 3D resembling the cone or can be cut in a layer view to see either a circle (*yz* plane) or a triangle with flattened tip (*xz* or *xy* plane).

The resolution phantom consists of five tubes with 1 mm radius filled with perimag of concentration 50 mmol(Fe)/L. The five tubes have a common origin in one point of the phantom. From that point they run in different directions in the *xy* and *yz* plane. While one tube points directly in *y* direction, the two tubes in the *yz* plane have an angle of 10° and 15° to the *y* axis. The two tubes in the *xy* plane have an angle of 20° and 30° to the *y* axis. The higher angles in the *yz* plane are due to the stronger gradient strength of the selection field in *z* direction, which results in higher spatial resolution. In order to determine the resolution of the reconstructed particle concentration one can consider *xz* planes and test if the tubes can be resolved. By varying the distance of the chosen plane to the origin of the tubes, the tube distance can be varied.

The last phantom is a concentration phantom consists of 8 cubes with 2 mm edge length and 8 μL volume. The cubes are placed at the corners of a cuboid with 12 mm distance in the *x* and *y* directions and 6 mm distance in the *z* direction. The cubes are numbered from 1 to 8 starting with the top layer on the front left (positive *x* and *y* directions), counting clockwise. Then starting with the lower layer with number 5 on the front left (positive *x* and *y* directions), counting clockwise. The numbers are also indicated in the CAD sketch in Fig. 2. The 8 cubes are filled with different concentrations starting from 100 mmol(Fe)/L and reducing the concentration by a factor of 1.5 from one to another cube. The concrete concentrations are listed in the following table. They are arranged in a way that the highest and the lowest concentration are not within the same plane.

**Table:** 

Number	1	2	3	4	5	6	7	8
Concentration [mmol(Fe)/L]	44.4	100	29.6	8.77	19.7	66.6	13.1	5.85

### 1.2 Sequences

Each phantom was measured with three different measurement sequences [3]. All three are 3D sequences, but the number of applied excitation directions is increased from one to three. In case of the 1D and 2D excitation, the dimension with no excitation is sampled by moving the phantom stepwise using a robot. For the 1D excitation this movement was first performed in the *z* direction and then in the *y* direction, i.e. *xz* slices were measured slice by slice. The following table summarizes the parameters of the three measurement sequences.

**Table:** 

Parameter	1D Cartesian	2D Lissajous	3D Lissajous
Drive-Field Amplitude	12 mT × 0 mT × 0 mT	12 mT × 12 mT × 0 mT	12 mT × 12 mT × 12 mT
Drive-Field Frequency	2.5/102 MHz × 2.5/96 MHz × 2.5/99 MHz		
Selection-Field Gradient	−1.0 T/m × −1.0 T/m × 2.0 T/m		
Repetition Time	40.8 μs	652.8 μs	21.54 ms
Number of Patches	1 × 19 × 19	1 × 1 × 19	1 × 1 × 1
Number of Periods per Patches	1000	1000	1
Number of Frames	1	1	1000

### 1.3 Phantom datasets

For each phantom and for each sequence a measurement was performed. The measurements are listed in the following table. The study name and the experiment number can uniquely identify them.

**Table:** 

Study	Experiment Number	Scanner	Sequence	Tracer
shapePhantom	1	Bruker	1D Cartesian	perimag
shapePhantom	2	Bruker	2D Lissajous	perimag
shapePhantom	3	Bruker	3D Lissajous	perimag
resolutionPhantom	1	Bruker	1D Cartesian	perimag
resolutionPhantom	2	Bruker	2D Lissajous	perimag
resolutionPhantom	3	Bruker	3D Lissajous	perimag
concentrationPhantom	1	Bruker	1D Cartesian	perimag
concentrationPhantom	2	Bruker	2D Lissajous	perimag
concentrationPhantom	3	Bruker	3D Lissajous	perimag

### 1.4 Calibration datasets

In addition to the object measurements that contain the data of the right hand side of the MPI imaging equation ***Sc*** = ***u***, OpenMPIData contains for each measurement sequence two calibration measurements that basically store the system matrix ***S***. All calibration scans are measured using a voxel-shaped delta sample of size 2 mm × 2 mm × 1 mm filled with perimag (concentration 100 mmol(Fe)/L). The delta sample is moved on a regular grid covering a volume of size 24 mm × 24 mm × 12 mm. While the first calibration scan is measured at 19 × 19 × 19 grid positions, the second calibration scan is measured at 37 × 37 × 37 grid positions.

**Table:** 

Study	Experiment Number	Grid	Scanner	Sequence	Tracer
calibration	1	19 × 19 × 19	Bruker	1D Cartesian	perimag
calibration	2	19 × 19 × 19	Bruker	2D Lissajous	perimag
calibration	3	19 × 19 × 19	Bruker	3D Lissajous	perimag
calibration	4	37 × 37 × 37	Bruker	1D Cartesian	perimag
calibration	5	37 × 37 × 37	Bruker	2D Lissajous	perimag
calibration	6	37 × 37 × 37	Bruker	3D Lissajous	perimag

## 2 Experimental design, materials, and methods

The OpenMPIData project has two different purposes. The first aim is to provide a fixed number of reference datasets that cover several challenging aspects for image reconstruction methods. These datasets are content of the present document and documented in detail. The second aim of the OpenMPIData project is to provide a platform where open MPI data can be hosted. In order to add new datasets to the platform one can create an issue at the Github project page.

The measurements discussed in this paper were measured in February 2018 using the preclinical MPI scanner (Bruker, Ettlingen) that was installed at the University Medical Center Hamburg-Eppendorf in 2014. All measurements were done with the equipped receive coils. The phantoms were designed with the CAD software SolidWorks and printed with a stereolithography 3D printer (Form2, Formlabs). The phantoms were then filled with perimag that was first diluted to the concentration given in the data section. The phantoms were mounted at a rod provided by the vendor of the MPI system and moved into the scanner center using a robot. Then, the measurements were performed using the system software ParaVision. Calibration measurements were done using the calibration method within the system software ParaVision. Instead of a phantom, the delta sample was mounted onto the rod that moves the sample into the scanner bore.

**Fig. 3:**
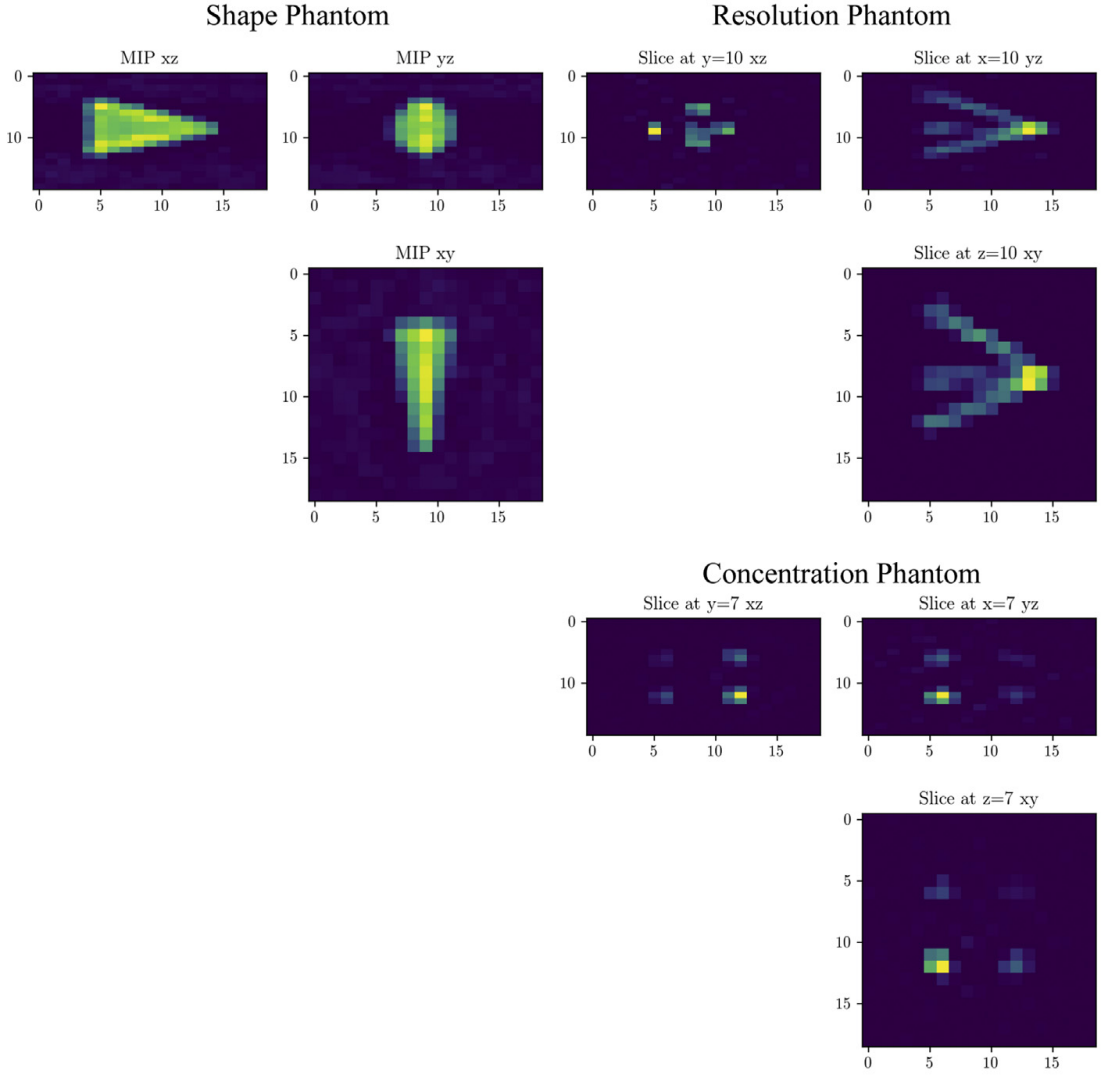
Baseline reconstruction results using the Kaczmarz algorithm for the shape phantom (top left), the resolution phantom (top right), and the concentration phantom (bottom right). In all cases the 3D Lissajous measurement was reconstructed. For the shape phantom maximum intensity projections (MIP) are shown.

After performing the measurements, all measurements were converted from the proprietary vendor data format into the open Magnetic Particle Imaging Data Format (MDF) [4] using the software package MPIFiles.jl [5]. The OpenMPIData Github repository contains example scripts that perform a baseline reconstruction and visualize the results. The baseline reconstruction is based on the software package MPIReco.jl [6]. Example reconstructions of all phantoms for the 3D Lissajous sequence are shown in Fig. 3.
